# 

**Published:** 1888-01-28

**Authors:** 


PRICE ONE PENNY.

[illegible]o. 70, Vol. III—January 28th, 1888.

[Registered at the General Post Office as a Newspaper.]

Per Annum, 6s. 6d., post free.

Monthly Parts, 6d.; post free, 7½d.

Ditto per Ann., 7s. 6d. post free.

### THE HOSPITAL

A Weekly Institutional Journal of

